# Fabrication of MoS_2_/rGO hybrids as electrocatalyst for water splitting applications†

**DOI:** 10.1039/d4ra00697f

**Published:** 2024-04-19

**Authors:** Muhammad Shahzeb Khan, Tayyaba Noor, Erum Pervaiz, Naseem Iqbal, Neelam Zaman

**Affiliations:** a School of Chemical and Materials Engineering (SCME), National University of Sciences and Technology (NUST) Islamabad 44000 Pakistan tayyaba.noor@scme.nust.edu.pk +92 51 90855121; b U.S.-Pakistan Center for Advanced Studies in Energy (USPCAS-E), National University of Sciences and Technology (NUST) Islamabad 44000 Pakistan

## Abstract

Environmental degradation and energy constraint are important risks to long-term sustainability in the modern world. Water splitting is a vital approach for environmentally friendly and sustainable energy storage, providing a clean way to produce hydrogen without pollutants. Preparing a catalyst that is active, bifunctional, and durable for water splitting is a difficult task. We addressed the difficulty by creating a bifunctional heterogeneous catalyst, MoS_2_/rGO, with an ideal weight percentage of 5 wt% by a hydrothermal process. The optimized sample showed exceptional electrocatalytic activity, requiring an overpotential of 242 mV and 120 mV to achieve a current density of 10 mA cm^−2^ in the Hydrogen Evolution Reaction (HER) and Oxygen Evolution Reaction (OER). Furthermore, our synthesized catalyst was validated for its exceptional water-splitting capacity, with the optimized sample showing low Tafel slope values of 59 mV dec^−1^ for HER and 171 mV dec^−1^ for OER. The significant OER and HER activity seen in the 5 wt% MoS_2_/rGO hybrid, compared to other hybrids, is due to the many catalytic active sites that aid in charge and electron transport, as well as the synergistic interaction between MoS_2_ and rGO.

## Abstract 2

Environmental degradation and energy constraint are important risks to long-term sustainability in the modern world.

## Graphical abstract

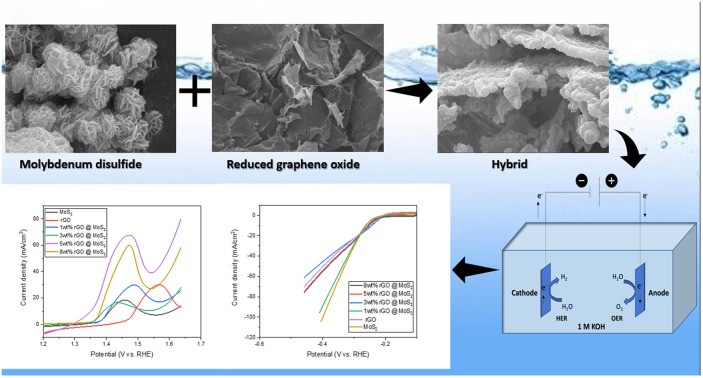

## 1. Introduction

Energy plays a significant role in day-to-day life and modern industry functioning. Throughout the last couple of decades, the vast majority of the world's energy requirements have been met through the consumption of fossil fuels.^1–3^ However, with an increase in the population at such a high rate, the energy requirement is also increasing at a massive rate resulting in an increased burden over the current fossil fuel reserves. Since fossil fuel reserves are finite, they cannot satisfy the world's growing energy needs. An increased consumption rate may result in the complete exhaustion of fossil fuels someday, resulting in a severe energy shortage problem in the future.^4–6^ According to studies, it is speculated that at the current pace of energy consumption, the existing fossil fuel reserves can only provide for 40 years of petroleum, 60 years of natural gas, and 156 years of coal, due to which their prices will continue to rise, as seen in 2008 when the fuel prices surged.^6,7^ At the same time, the consumption of fossil fuels leads to environmental problems because they generate harmful gases, including CO_2_, SO_*x*_, and NO_*x*_ resulting in the greenhouse effects and adding to global warming.^8,9^

Considering these issues, studies have been conducted recently to find alternate energy sources to ensure future world stability.^10^ Some of the most important factors to think about for an alternate sustainable energy source are energy security and supply, air quality and health improvement, economic competitiveness, greenhouse gas reduction.^11–13^ In this regard, it has been discovered that hydrogen may be the perfect sustainable energy source for the future, pertaining to its excellent properties, even though it is not available as a ready-to-use substance.^12,14,15^ Hydrogen production now involves various techniques, including methane steam reforming, partial oxidation, coal gasification, but these methods utilize fossil fuels, so they do not fulfill the idea of sustainable and clean hydrogen production.^16,17^ To mitigate this issue, the electrochemical splitting of water has been regarded as an effective, environmentally friendly, and scalable method, and it can be used to produce hydrogen on a big scale. However, it is the slow kinetics of the hydrogen evolution reaction (HER) as well as the oxygen evolution reaction (OER) that restrict the output efficiency of electrochemical water splitting, resulting in an increase in the cost of process.^18–21^ Even though noble metal-derived catalysts like Pt, Ir, and Ru-derived electrocatalysts have demonstrated promising electrocatalytic capabilities for water splitting applications, their broad application is constrained by their severe scarcity and very high price.^22–26^ As a result, it is of the utmost significance to design a catalyst for water splitting that is both inexpensive and plentiful in the earth to make the process more efficient, economical, and sustainable.

Recently, transition metals and their hybrids have been the subject of research for their potential application as electrocatalysts in water splitting reactions. This is attributable to the vacant d-orbital of transition metals and their hybrids, which plays an essential part in forming a moderate hydrogen bond and is highly significant for hydrogen generation.^27,28^ In this particular instance, there has been an emphasis placed on the use of transition metal compounds, particularly transition metal sulfides that have been found to possess high activity for HER and OER because of their unique electrical characteristics, high valence, transition metal ion synergy, and great corrosion resistance in an alkaline environment.^29–31^ Molybdenum disulfide (MoS_2_) is a robust substance having a distinctive two-dimensional layered architecture similar to graphene. It is known as a flexible material having a number of possible uses, such as electronic devices,^32,33^ lithium-ion batteries,^34^ and HER catalysts.^35,36^ Owing to their outstanding electrocatalytic capabilities, there has been a lot of research done on MoS_2_-based materials both experimentally and computationally since Sobczynski identified MoS_2_ as an effective HER electrocatalyst in 1991.^37^ According to Norskov's group, the hydrogen binding energy of MoS_2_ is quite comparable to that of Pt.^38^ Surprisingly, as far as HER is concerned, the electrocatalytic capabilities arise from atoms that are present in the edge regions of MoS_2_. At the same time, the catalytic activity in the MoS_2_ basal area is nonexistent. As a result of the increased amount of active sites exposed at the point where Sulphur is present, MoS_2_ nanoparticles have a significantly higher activity level over bulk MoS_2_.^39^ Coupling of MoS_2_ with other conductive supports has been shown to greatly improve the electrocatalytic activity as opposed to bulk MoS_2_ owing to the improved electronic structure, increase in surface area, and stability.^40,41^ Nevertheless, the electrical conductivity of MoS_2_ is weak, despite the fact that it is a critical element in overall electrocatalytic efficiency. It has come to light, and some suggestions have been made regarding various methods that can be utilized to boost the electrocatalytic activity of catalysts based on MoS_2_. One such method is to use carbon-based materials as support for the MoS_2_ in an attempt to achieve a maximum number of active sites that are exposed while preventing the MoS_2_ nanocomposites from aggregating. In this regard, Cao *et al.* synthesized MoS_2_ nanosheets (MoS_2_ NS) supported on multi-walled carbon nanotubes (MWCNTs) following a hydrothermal route resulting in MoS_2_ NS/MWCNT with outstanding HER activity and excellent cyclic stability. In this method, surface functionalized MWCNTs were first synthesized by utilizing nitric acid (HNO_3_) solution, introducing oxygenated groups, and increasing the hydrophilicity. It was then followed by the preparation of a hybrid of MoS_2_ NS/MWCNT through the reaction of sodium molybdate and thiourea, along with the functionalized MWCNTs. The exceptional catalytic performance resulted from the conductive MWCNT substrate and the evenly distributed metallic 1T phase of the MoS_2_ nanosheets, which is highlighted by the occurrence of numerous active edges and the efficient electron transport channel.^42^ Pang *et al.* in their study, utilized carbon fibers (CF) as support to synthesize a hybrid with cobalt oxide (CoO) decorated MoS_2_ by following a two-step method consisting of both hydrothermal process as well as electrochemical deposition. The electrocatalytic effectiveness of the developed catalyst was examined in an alkaline medium, and it was shown to have excellent HER and OER activities owing to the enhanced amount of active sites, improved structural properties, and the synergetic interplay between the different components of the hybrid.^43^ Ren *et al.* synthesized a hybrid of phosphorus-doped with molybdenum sulfide (P–MoS_2_) following a post-doping synthesis approach. In this approach, triphenylphosphine (C_18_H_15_P) was used to synthesize P–MoS_2_ nanosheets following a facile annealing method inside a tube furnace under the Ar/H_2_ environment. In their method, phosphorus was chosen as a dopant as the Mo–P bond was found to aid in the HER performance by elevating both the conductivity and the amount of active edge sites of the MoS_2_ nanosheets.^44^

Herein, we developed a bifunctional heterogeneous catalyst MoS_2_/rGO with an optimal weight percentage of 5 wt% using a hydrothermal method. The optimized sample demonstrated extremely strong electrocatalytic activity having an overpotential of 242 mV at 10 mA cm^−2^ for the HER. For OER, the hybrid displayed an exceptionally small overpotential of about 120 mV to drive the current density of 10 mA cm^−2^. Remarkable OER and HER activity of 5 wt% MoS_2_/rGO among other hybrids is attributable to an immense amount of the suitable catalyst active sites that facilitate charge and electron transport as well as the synergistic effect between MoS_2_ and rGO.

## 2. Experimental section

### 2.1. Materials

In the course of our research, we made use of chemicals and reagents of an analytical grade without doing any additional purification. The main chemicals required comprising ammonium molybdate ((NH_4_)_6_Mo_7_O_24_·4H_2_O), thiourea (CH_4_N_2_S), graphite powder, sodium nitrate (NaNO_3_), potassium permanganate (KMnO_4_) and hydrogen peroxide (H_2_O_2_) and were purchased from Sigma Aldrich whereas deionized (DI) water, sulfuric acid (H_2_SO_4_), hydrazine hydrate (N_2_H_4_) and ethanol were secured from Merck.

### 2.2. Synthesis of graphene oxide and reduced graphene oxide

In our study, graphene oxide (GO) was successfully synthesized by following Hummers' method, while the synthesis of reduced graphene oxide (rGO) has been accomplished by chemically reducing the already synthesized GO using Hydrazine hydrate in a reflux approach for 24 h at temperature as high as 100 °C.^45,46^

### 2.3. Synthesis of pure molybdenum disulfide (MoS_2_)

Typically, 0.75 g AHM and 5.1 g thiourea were mixed in 50 mL DI water, followed by stirring, so the solution became transparent and clear. The resulting solution was then placed inside a stainless steel autoclave fitted with a 100 mL PTFE liner and kept at a temperature of about 200 °C for 24 h. After getting MoS_2_, it was first washed with DI water, followed by ethanol, before being placed inside an oven to dry overnight at 70 °C.

### 2.4. Synthesis of MoS_2_/rGO hybrids

**Fig. 1:**
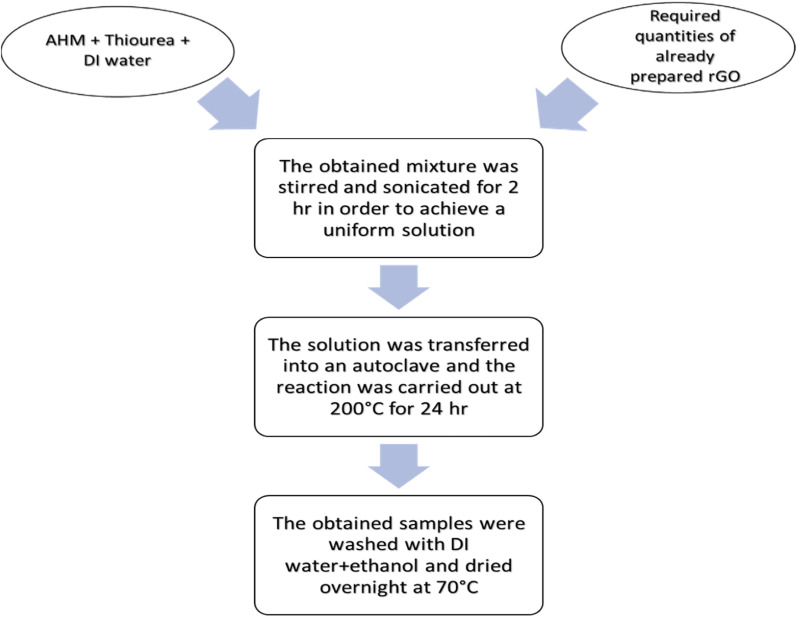
Schematic of 1, 3, 5, 8 wt% MoS_2_/rGO hybrids.

Hybrids of MoS_2_ and rGO were synthesized in weight ratios of 1, 3, 5, and 8 wt% following a hydrothermal approach. In this method, 0.75 g AHM and 5.1 g thiourea were mixed in 50 mL DI water, followed by stirring to achieve a consistent and transparent solution. To synthesize hybrids of different weight ratios, already prepared rGO was added to the solution with 0.0585 g, 0.1755 g, 0.2925 g, and 0.468 g for 1, 3, 5, and 8 wt%, respectively. For the purpose of perfectly mixing the rGO with the mixture, the mixture was subjected to sonication for 2 h until a black solution was achieved. Following this, the solution that had been produced was shifted to a stainless steel autoclave with a 100 mL PTFE liner and then put inside an oven at about 200 °C to carry out the reaction for 24 h. The obtained hybrids were then subjected to washing with DI water followed by ethanol and kept in an oven for drying overnight at 70 °C (Fig. 1).

### 2.5. Characterization

The samples under investigation have been examined *via* X-ray diffraction (STOE-Seifert X'Pert PRO), utilizing values of 2*θ* in the range of 0° to 90° making use of Cu-Kα radiation. For the further morphological study of the synthesized samples, scanning electron microscopy (JEOL-instrument JSM-6490A) was used coupled with EDX. Further, the presence of functional groups in the prepared material is checked by Fourier Transform-infrared (FT-IR).

## 3. Results and discussion

**Fig. 2:**
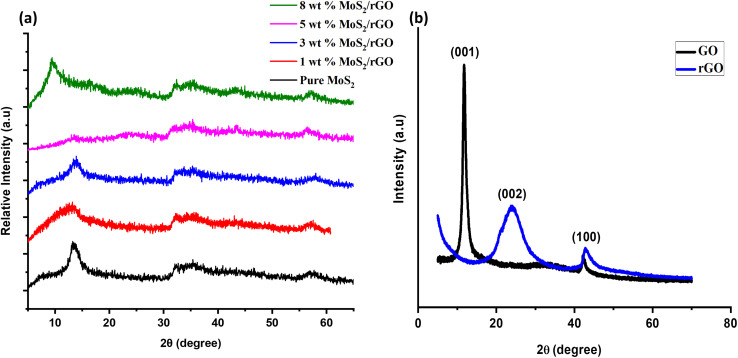
XRD analysis of (a) pure MoS_2_ and 1, 3, 5, 8 wt% MoS_2_/rGO hybrids, (b) rGO and GO.

The crystalline nature and structural properties of the synthesized catalyst samples were examined by utilizing XRD. Considering the pure MoS_2_, the peaks observed at ∼14.2°, ∼33.0°, and ∼58.9° were attributed to the (002), (100), and (110) planes, respectively.^47^ In the case of pure GO and rGO, the strong characteristic peak at ∼10.5° corresponds to the successful synthesis of GO, and the peak at ∼25.4° corresponds to the (002) plane of rGO. The peak at ∼43.5° represents the presence of non-exfoliated graphite.^48^ Considering the hybrids of MoS_2_/rGO, especially the 8 wt% MoS_2_/rGO, there is a shift in the (002) plane from ∼14.2° (interlayer spacing of 6.2 Å) to ∼9.5° (interlayer spacing of about 9.2 Å) as compared to other hybrids and pure MoS_2_. This might be a result of the disorder engineering of MoS_2_ nanosheets giving rise to the increased interlayer spacing. This increased interlayer spacing has been found to be a result of incorporating the reaction agent or other derivatives into the two layers of S–Mo–S.^49,50^ Moreover, the homogeneous dispersion of rGO sheets and successful coating with MoS_2_ nanosheets in the 5 wt% hybrid might contribute to the alteration in the XRD pattern. The absence of the (002) peak in this case suggests that the interlayer spacing changes in a distinct manner, potentially influenced by the specific ratio of MoS_2_ to rGO. This alteration could be due to a more intimate interaction between MoS_2_ and rGO at the 5 wt% loading, leading to variations in the crystallographic features observed in the XRD pattern.^51,52^ The sample features broad peaks, which are characteristic of tiny crystallites and can be used to identify them. The Scherrer equation (eqn (1)) was used to determine the average grain size of MoS_2_ particles from the lengths of the diffraction peaks, providing conclusive evidence.1
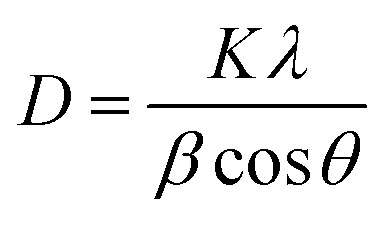
where, *D* = crystallite size (nm), *K* = 0.9 (Scherrer constant), *λ* = 0.15406 nm (wavelength of X-ray sources). Using this equation, the crystal size of the pure MoS_2_ was calculated to have a value of 15.53 nm. These results demonstrated that the required hybrids of different wt% have been synthesized successfully (Fig. 2).

**Fig. 3:**
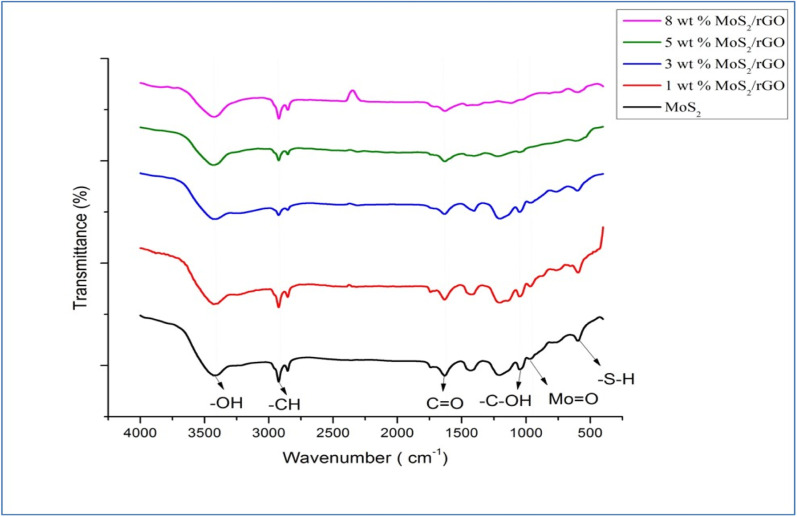
FTIR spectra of prepared samples.

We may learn more about the sample's chemical composition and synthesis method using FTIR analysis, which is performed to efficiently identify the sample by exploring the functional categories included in it. Fig. 3 displays FTIR spectra of the various synthesized samples, including pure MoS_2_, rGO, and the 1, 3, 5, and 8 wt% MoS_2_/rGO hybrids. The rGO spectra reveal that the water molecules are distorted in bands around 1630.44 cm^−1^. The occurrence of a broad peak at approximately 3438.61 cm^−1^ has been attributed to a number of possible explanations, one of which is the presence of skeletal vibrations associated with unoxidized graphitic domains, while another possibility is that it is caused by the vibrations of water molecules that have been adsorbed. Further, the peak corresponding to the –CH bond can be found at approximately 2900 cm^−1^, while the peak representing the C=O bond is located at about 1600 cm^−1^. The peak observed at approximately 2300 cm^−1^ in Fig. 3 of the FTIR spectrum, it is attributed to the stretching and bending of O–Mo vibrations. This observation suggests the involvement of oxygen–molybdenum interactions in the sample, which can significantly influence the spectral features in this region. In addition, the Mo=O bond is responsible for the peak at roughly 908 cm^−1^, while the –SH bond is represented by the peak at around 660 cm^−1^. The wavenumber range that encompasses 400 to 1000 cm^−1^ is linked to the creation of the S–S bond.

The structural and compositional characteristics of the synthesized material were evaluated using a scanning electron microscope (SEM). Fig. S1† displays the SEM images of pure MoS_2_, rGO, and the synthesized 1, 3, 5, and 8 wt% MoS_2_/rGO hybrids.

It can be seen that the produced MoS_2_ possesses a nanoflower-like structure made of transparent nanoflakes. After being subjected to hydrothermal conditions for 24 h, the material forms uniformly sized nanoflakes, which coalesce into a relatively open and deep porous nanostructure that facilitates rapid electron transport. Since only thin MoS_2_ nanosheets are created in the absence of a 2D structured support like rGO, the formation of substantial 2D structures in the pictures as mentioned earlier indicates that the MoS_2_ nanosheets have evenly grown on the 2D structured rGO sheets. This also explains how rGO may keep MoS_2_ from clumping together by giving it structural stability. The MoS_2_ shell is composed of quite small nanosheets of a few nanometers in perpendicular configuration, as seen in high-magnification SEM images, and there are many empty spaces between the nanosheets. In hybrid, the surface of the materials increases because of the presence of rGO. The greater surface area means that the number of active sites available for the reactions will increase and more electrons will pass easily within the catalyst. In this way the efficiency of the hybrid is increased, and it shows higher activity as compared to the pure MoS_2_ and pure rGO.

Energy-dispersive X-ray spectroscopy is an additional method of characterization that is utilized for the purpose of doing elemental analysis (EDX). As indicated in the table, it suggests that the created hybrid material primarily consists of carbon, oxygen, sulfur, and molybdenum. It can be deduced from this that a hybrid of MoS_2_ and rGO has been successfully developed. The high concentrations of carbon and oxygen suggest the presence of rGO, while the concentrations of molybdenum and sulfur represent the presence of MoS_2_ in the hybrid (Table 1).

**Table 1:** EDX study of synthesized hybrids

Elements	1 wt% MoS_2_/rGO	3 wt% MoS_2_/rGO	5 wt% MoS_2_/rGO	8 wt% MoS_2_/rGO
C	16.9	28.6	43.45	44.88
O	22.9	6.8	8.9	9.12
S	8.4	22.1	17.65	8.95
Mo	51.9	22.0	30	37.05

### 3.1. Electrochemical measurements

The electrochemical investigation was entirely carried out on a 3-electrode assembly utilizing the prepared Ni foam electrodes as working electrodes, the Pt wire served as the auxiliary electrode, and the Ag/AgCl electrode served as the reference electrode, respectively. The catalytic activity of all the produced samples was studied in 1 M alkaline solution at varied scan rates. The stability tests were carried out through chronopotentiometry analysis for about 7200 s. The electrochemical impedance spectroscopy (EIS) was performed with a frequency range of 205 Hz to 0.1 Hz and by applying an AC voltage of 10 mV.

#### 3.1.1. Preparation of MoS_2_ and its rGO based MoS_2_ electrodes

For electrochemical analysis, firstly, all the sample ink was synthesized by dissolving 3 mg of our prepared catalyst in a 3 : 97 μl mixture of Nafion and ethanol, respectively, followed by sonication for 4 h. The as-prepared ink was then deposited on Nickle foam cut into 1 × 1 pieces, and dried overnight at about 70 °C.

#### 3.1.2. Hydrogen evolution reaction (HER)

**Fig. 4:**
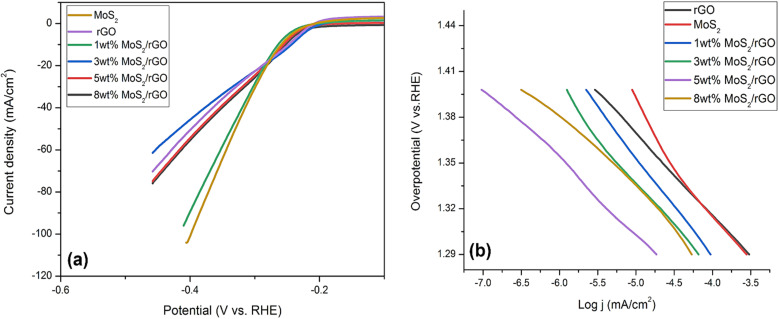
(a) LSV curves HER for pure MoS_2_, rGO, 1, 3, 5 and 8 wt% hybrids (b) corresponding Tafel slopes of pure MoS_2_, rGO, 1, 3, 5 and 8 wt% hybrids.

A three-electrode setup was used in a 1 M alkaline solution for the purpose of investigating on the electrochemical properties of pure MoS_2_, rGO, and related hybrids. In comparison to pure MoS_2_ and rGO, the hybrids showed higher HER activity. Due to Pt's superior catalytic ability for HER, pure MoS_2_ and rGO hybrids require enhancements in activity to meet industry standards. A representation of how linear sweep voltammetry curves are employed to assess the HER activity of the samples in an alkaline solution is provided in Fig. 4. Pure MoS_2_ and rGO were found to exhibit overpotential values of 258 mV and 264 mV, respectively, to drive a current density of 10 mA cm^−2^. Likewise, MoS_2_ hybrids with rGO had distinct overpotentials. Hybrids with wt% of 1, 3, 5, and 8 had overpotential values of 256 mV, 252 mV, 242 mV, and 250 mV, respectively. Among the hybrids under investigation, the one exhibiting an overpotential of just 242 mV at 10 mA cm^−2^ was the one with which the 5 wt% fraction was associated. The electrocatalyst performance and kinetics of the materials for HER were investigated by calculating their Tafel slopes using the Tafel equation.^53^*η* = *b* log *j* + *a*Where *a* is Tafel constant, *b* is Tafel slope, and *j* is current density.

As a rule of thumb, a steeper Tafel slope indicates a more active synthesized catalyst regarding HER. The Tafel slope of 5 wt% hybrid was around 59 mV dec^−1^, that was significantly less compared to the other synthesized catalysts employed in this investigation (Fig. 4a). Pure MoS_2_ had a Tafel slope of 75 mV dec^−1^, whereas 1 wt% MoS_2_/rGO had a slope of 70 mV dec^−1^, 3 wt% MoS_2_/rGO had a slope of 67 mV dec^−1^, and 8 wt% MoS_2_/rGO had a Tafel slope value of 66 mV dec^−1^. Additionally, the different kinetics of the 5 wt% MoS_2_/rGO hybrid as compared to the other electrocatalysts were shown by the reduced Tafel slope for the 5 wt% MoS_2_/rGO hybrid as illustrated in Fig. 4b. Following are the kinetic stages involved in the Volmer–Heyrovsky reaction:H_2_O + M + e^−^ → M − H_ads_ + OH^−^H_2_O + M − H_ads_ + e^−^ → H_2_ + OH^−^In this case, M stands for “working sites.” Adsorbed hydrogen is converted into an intermediate product when water molecules from the alkaline solution are released on the catalyst surface. The high surface area of MoS_2_ hybrids has been shown in previous research to make them effective water dissociation controllers, making them a good fit for the HER process in an alkaline environment. The impressive behavior of MoS_2_/rGO hybrids, particularly 5 wt% MoS_2_/rGO, can be ascribed to the mutual effect that MoS_2_ and rGO have on one another. In addition, the conductive support that the Ni foam provides is also a contributing factor. Therefore, the electrocatalyst enhanced its performance in terms of hydrogen adsorption. In addition to offering adequate mechanical stability, Ni foam also creates a tight channel that permits water molecules to be delivered to the active sites. The improved availability of MoS_2_ nanoparticles to active sites was the cause of the enhanced HER performance of the hybrids (Table 2).

**Table 2:** HER performance of different catalysts

S. no.	Samples	Target reaction	Overpotential (mV)	Tafel slope (mV dec^−1^)	Ref.
1	5 wt% MoS_2_/rGO	HER	242	59	This work
2	MoS_2_/NiS	HER	244	97	54
3	CoS_1.097_/MoS_2_	HER	249	75	55
4	MoS_2_ NDs/VS_2_	HER	291	58.1	56
5	Co_9_S_8_/NC@MoS_2_	HER	261	126.1	57
6	Platinum wire	HER	74.8	—	58

#### 3.1.3. Oxygen evolution reaction (OER)

**Fig. 5:**
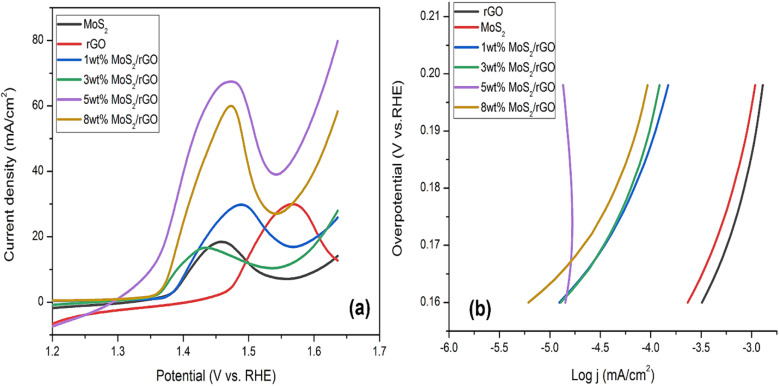
(a) LSV curves OER for pure MoS_2_, rGO, 1, 3, 5 and 8 wt% hybrids (b) corresponding Tafel slopes of pure MoS_2_, rGO, 1, 3, 5 and 8 wt% hybrids.

Using LSV polarization curves, we studied the electrocatalytic activity of the synthesized hybrids in 1 M alkaline solution in terms of OER. In contrast to the other catalysts that were prepared, the 5 wt% MoS_2_/rGO has displayed a significant improvement in OER performance since it is able to rapidly increase its current density at a potential of 1.55 V. The presence of this peak and its alignment with the oxidation behavior of MoS_2_ and rGO is indicative of the catalyst's unique electrochemical properties, contributing to its enhanced OER performance. Further, using the anodic current density of 10 mA cm^−2^, the overpotential of the 5 wt% MoS_2_/rGO hybrid was exceptionally low at 120 mV. Compared to pure MoS_2_, rGO, 1, 3, and 8 wt% MoS_2_/rGO hybrid, *i.e.*, 179 mV, 259 mV, 176 mV, 159 mV, and 150 mV, respectively, this had a significantly lower overpotential as shown in Fig. 5a.

The lower value of the Tafel slope of around 171 mV dec^−1^ allowed for a thorough investigation of the electrochemical properties of 5 wt% MoS_2_/rGO. Tafel slope of 257 mV dec^−1^ was measured for pure MoS_2_, 266 mV dec^−1^ was measured for rGO, and 196 mV dec^−1^ was measured for 1 wt% rGO. For 3 wt% MoS_2_/rGO, the slope was 195 mV dec^−1^. The Tafel slope value was 188 mV dec^−1^ for 8 wt% MoS_2_/rGO. The Tafel value of pure MoS_2_ represents the sluggish rate of the adsorption phenomenon of OH ions over the surface of the catalyst as given in Fig. 5b. The presence of rGO improves the OER kinetics by decreasing the Tafel value. Tafel values that were sufficiently low suggested high OER activity when contrasted with other commonly used catalysts. The small Tafel slope of 5 wt% hybrid indicated that the activity is a 4-electron process. An increase in potential enhances electrocatalytic performance by increasing the catalyst rate. This notable trait is generated by the interplay between absorbed oxygen species and transition metal cation sites. The oxidation of the MoS_2_/rGO hybrid was observed in four primary phases (Table 3).M + OH^−^ → M − OH^−^ + e^−^M − OH^−^ + OH^−^ → M − O + H_2_O + e^−^M − O + OH^−^ → M − OOH + e^−^M − OOH + OH^−^ → M + H_2_O + e^−^ + O_2_where M stands for the catalyst. In the OER polarization curves.

**Table 3:** OER performance of pure MoS_2_, rGO, 1, 3, 5 and 8 wt% hybrids

S. no.	Samples	Target reaction	Overpotential (mV)	Tafel slope (mV dec^−1^)	Ref.
1	5 wt% MoS_2_/rGO	OER	120	171	This work
2	Cu–Ru–MoS_2_	OER	308	50	59
3	Meso-Fe-MoS_2_/CoMo_2_S_4_	OER	290	65	60
4	Ni–Mo–S@CC	OER	320	88	61
5	MoS_2_–NiS_2_/NGF/NF	OER	235	71	62
6	IrO_*x*_	OER	250	—	63

**Fig. 6:**
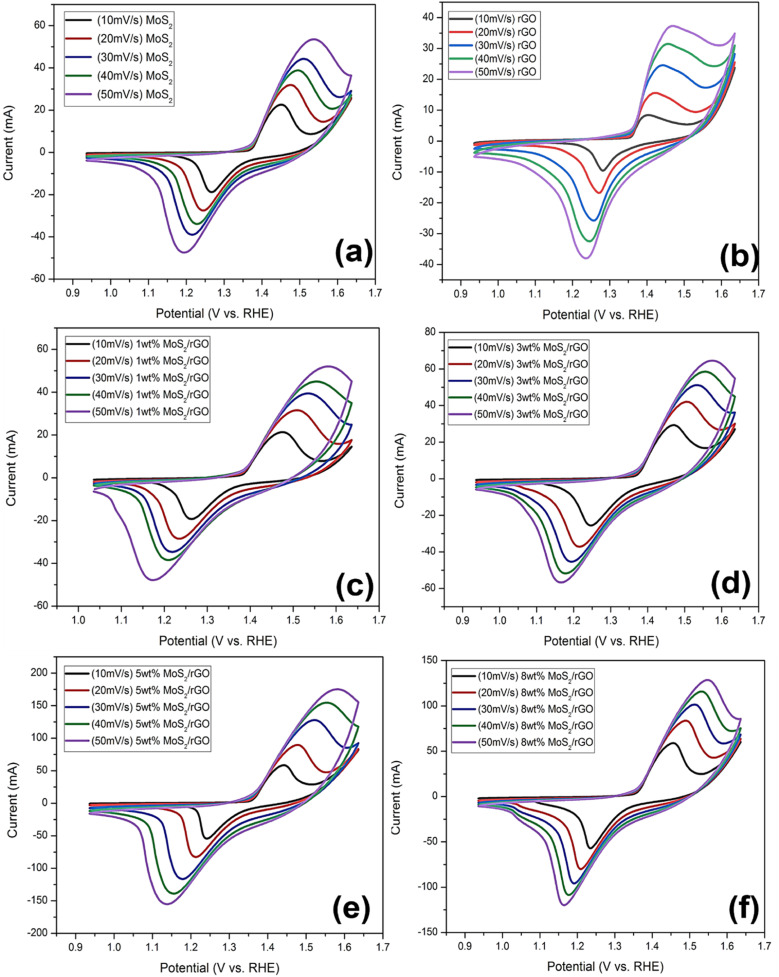
Cyclic voltammetry of (a) MoS_2_, (b) rGO, (c) 1 wt% MoS_2_/rGO, (d) 3 wt% MoS_2_/rGO, (e) 5 wt% MoS_2_/rGO, (f) 8 wt% MoS_2_/rGO at the scan rate of 10, 20, 30, 40 and 50 mV s^−1^.

Cyclic voltammograms were acquired from several samples scanned at voltage rates in the range of 10 to 50 mV s^−1^. There were two different redox peaks that can be noticed in the potential range of 0.9 to 1.7 V as shown in Fig. 6. The CV maintained its form even when the scan rate increased, a property that is indicative of the catalyst's exceptional cyclic stability, low resistance, and high electrocatalytic efficiency. In order to get a high current density, increasing the scan rate reduced the resistance of the diffusion layer. The stability of the produced catalysts has been demonstrated by the progressive growth of the redox peaks in response to increasing scan rates.

Among all the prepared rGO based hybrids, the enhanced catalytic performance for OER and HER observed in the reduced graphene oxide (rGO) composite containing 5 wt% rGO can be ascribed to a number of significant factors. First, the 5 wt% loading achieves a balance, ensuring the maximum number of active sites for catalytic reactions. This loading provides enough surface area and dispersion of rGO inside the composite, allowing for effective interaction with other catalytic components and efficient electron transfer during reactions. Second, this composition may also result in synergistic effects between rGO and other components, resulting in improved overall catalysis performance. Thirdly, loading of 5 wt% rGO not only preserves structural integrity within the composite, but also prevents agglomeration or structural flaws that might impair catalytic performance.

In addition to Ni foam role in providing essential mechanical stability, it also serves as a crucial conductive support in our catalyst system. As the unique structural feature of Ni foam includes channels that play a pivotal role in directing and delivering water molecules to the active catalytic sites. These channels act as conduits for the reactants, ensuring a well-organized and controlled environment for the electrochemical processes to take place. Thus, the Ni foam, with its multifaceted contributions, enhances the overall performance of the catalyst by promoting both mechanical stability and effective reactant transport to the active sites.^64^

Using cyclic voltammetry (CV) in 1 M KOH solution, the electrochemical active surface area (ECSA) of prepared pure MoS_2_, 1 wt% MoS_2_/rGO, 3 wt% MoS_2_/rGO, 5 wt% MoS_2_/rGO, and 8 wt% MoS_2_/rGO was determined. To determine double layer capacitance (*C*_dL_) of catalyst loaded electrode surface, current density data for all samples were linearly fitted concerning applied scan rates (as shown in Fig. 3 and 4 ESI file†). Slopes of linear fits applied on current density are regarded as *C*_dL_. The ECSA is then determined by formula given below:^62^ECSA = *C*_dl_/*C*_s_

*C*_s_ is the individual capacitance and is defined as the capacitance of an ideal flat surface catalyst. In general, McCrory reports that an average value of 40 F cm^−2^ is often used as *C*_s_ in alkaline solutions.^65^ Overall, ECSA values are calculated and given in Fig. S4 of the ESI files.† Among all prepared hybrids 5 wt% MoS_2_/rGO shows the high electrochemical surface area, Moreover, in 8 wt% MoS_2_/rGO the electrochemical surface area is lower as compared to 5 wt% which explains the agglomeration of support material when it is used in higher amount, as already explained in cyclic voltammetry of prepared catalysts.^63^

**Fig. 7:**
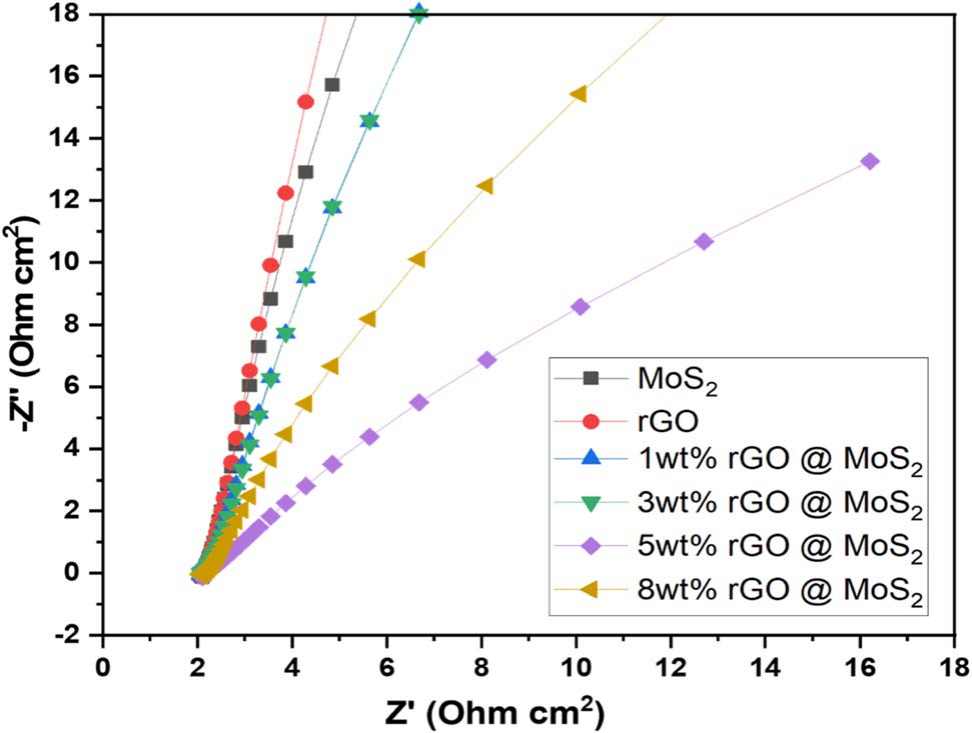
EIS analysis for pure MoS_2_, rGO, 1, 3, 5 and 8 wt% hybrids.

Further, the EIS results of synthesized catalysts were obtained by performing the experiment in a solution containing 1 M KOH. The frequency range used for the experiment was between 205 Hz and 0.1 Hz. The primary objective of EIS was to identify resistance. The Nyquist plot, also known as the EIS plot, is a diagram consisting of a semicircle and is used to determine the resistance resulting from a faradaic reaction at the surface of an electrode as shown in Fig. 7. Moreover, Randle circuit is employed to analyze experimental impedance data given in Table 4. This approach allowed us to recognize the various resistive and capacitive components within the system, providing valuable insights into its electrochemical dynamics. By incorporating these elements, the Randle circuit provides a comprehensive framework for understanding electrochemical behavior at the electrode–electrolyte interface. Further, determining charge transfer kinetics involves assessing the speed at which electrons migrate across the electrode surface during electrochemical reactions. It was evident, that 5 wt% MoS_2_/rGO possessed a low charge transfer resistance (*R*_ct_) of 5.072 × 10^−3^ Ω. This resistance was indicated by the shorter diameter of the semicircle, which demonstrated how effectively the charge could be moved. It has been determined that the polarization resistance of MoS_2_ was 19.58 × 10^−3^ Ω, while the polarization resistance of rGO was 24.53 × 10^−3^ Ω. The electrolyte resistance (Ru) of the MoS_2_, rGO, 1 wt% MoS_2_/rGO, 3 wt% MoS_2_/rGO, 5 wt% MoS_2_/rGO, and 8 wt% MoS_2_/rGO was determined to be 2.674, 1.920, 1.039, 2.210, 1.039, and 2.169 Ω respectively. Further, the reduction in the radius of the semicircle was indicative of a lower resistance value, which in turn demonstrated the capability of the catalyst to have a high charge transfer allowing it to perform efficiently.

**Table 4:** EIS data of the prepared samples

Sr. no.	Catalysts	*R*_ct_ (Ω)	Ru (Ω)	*C*_f_ (farad)
1	MoS_2_	9.58 × 10^−3^	1.034	2.210 × 10^−9^
2	rGO	24.53 × 10^−3^	1.920	116.3 × 10^−9^
3	1 wt% MoS_2_/rGO	9.606 × 10^−3^	1.039	2.211 × 10^−9^
4	3 wt% MoS_2_/rGO	7.609 × 10^−3^	2.210	1.769 × 10^−9^
5	5 wt% MoS_2_/rGO	5.072 × 10^−3^	1.039	3.252 × 10^−9^
6	8 wt% MoS_2_/rGO	5.912 × 10^−3^	2.169	2.766 × 10^−9^

**Fig. 8:**
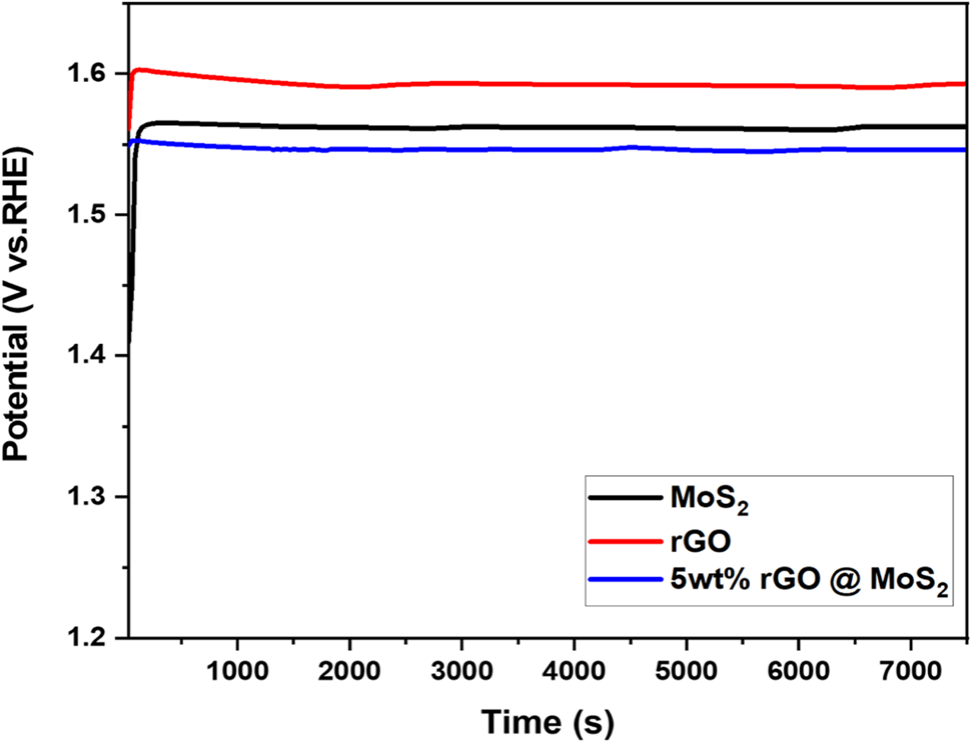
Chronopotentiometry of MoS_2_, rGO and 5 wt% MoS_2_/rGO.

The stability of the catalyst is critical for the adoption of hydrogen production technology as a fuel. In this regard, pure MoS_2_, rGO, and 5 wt% MoS_2_/rGO were then subjected to a stability test at 10 mA for 7200 s after performing 1000 cycles, as indicated in Fig. 8. According to the chronopotentiometry analysis, the catalyst displayed a stable behavior, which was attributed to its structural features. After 7200 s hour, the potential reacted quickly but consistently. This occurred as a consequence of the fact that the synthesized hybrid becomes activated during evolution.

## 4. Conclusions

In summary, we developed a bifunctional heterogenous catalyst MoS_2_/rGO with an optimal weight percentage of 5 wt%. The sample was prepared successfully using the hydrothermal technique, using thiourea as the sulfur source and AHM as the molybdenum source. The efficient movement of electrons in such a hybrid is due to a special synergistic effect. At a current density of 10 mA cm^−2^, the optimized sample had exceptionally high levels of both HER and OER activity, exhibiting very small overpotentials of 242 mV and 120 mV, respectively. The extraordinary HER and OER activity of hybrid 5 wt% MoS_2_/rGO, in comparison to that of other hybrids may be ascribed to the presence of a significant number of active sites which enable the movement of electrons and charges. Because of the catalyst's remarkable stability and durability, it has the potential to be useful in a variety of practical applications. This research lays the way for the creation of bifunctional materials which may be utilized in the overall water splitting process. As a result, additional research into design techniques and reaction mechanisms is necessary in order to create a better and more active catalyst that is also practicable.

## Conflicts of interest

The authors declare that they have no known competing financial interests or personal interests that could have appeared to influence the work reported in this paper.

## Supplementary Material

RA-014-D4RA00697F-s001
