# Supplementary material for: Fabrication of MoS2/rGO hybrids as electrocatalyst for water splitting applications
Source: RSC Adv. 2024 Apr 19;14(18):12742–53. doi: 10.1039/d4ra00697f (PMC11027038; doi:10.1039/d4ra00697f)
Supplement: RA-014-D4RA00697F-s001 [file RA-014-D4RA00697F-s001.pdf]

Electronic Supplementary Material (ESI) for RSC Advances.
This journal is © The Royal Society of Chemistry 2024

Supplimetary data file

Fabrication of MoS_2/rGO hybrids as electrocatalyst for water splitting applications

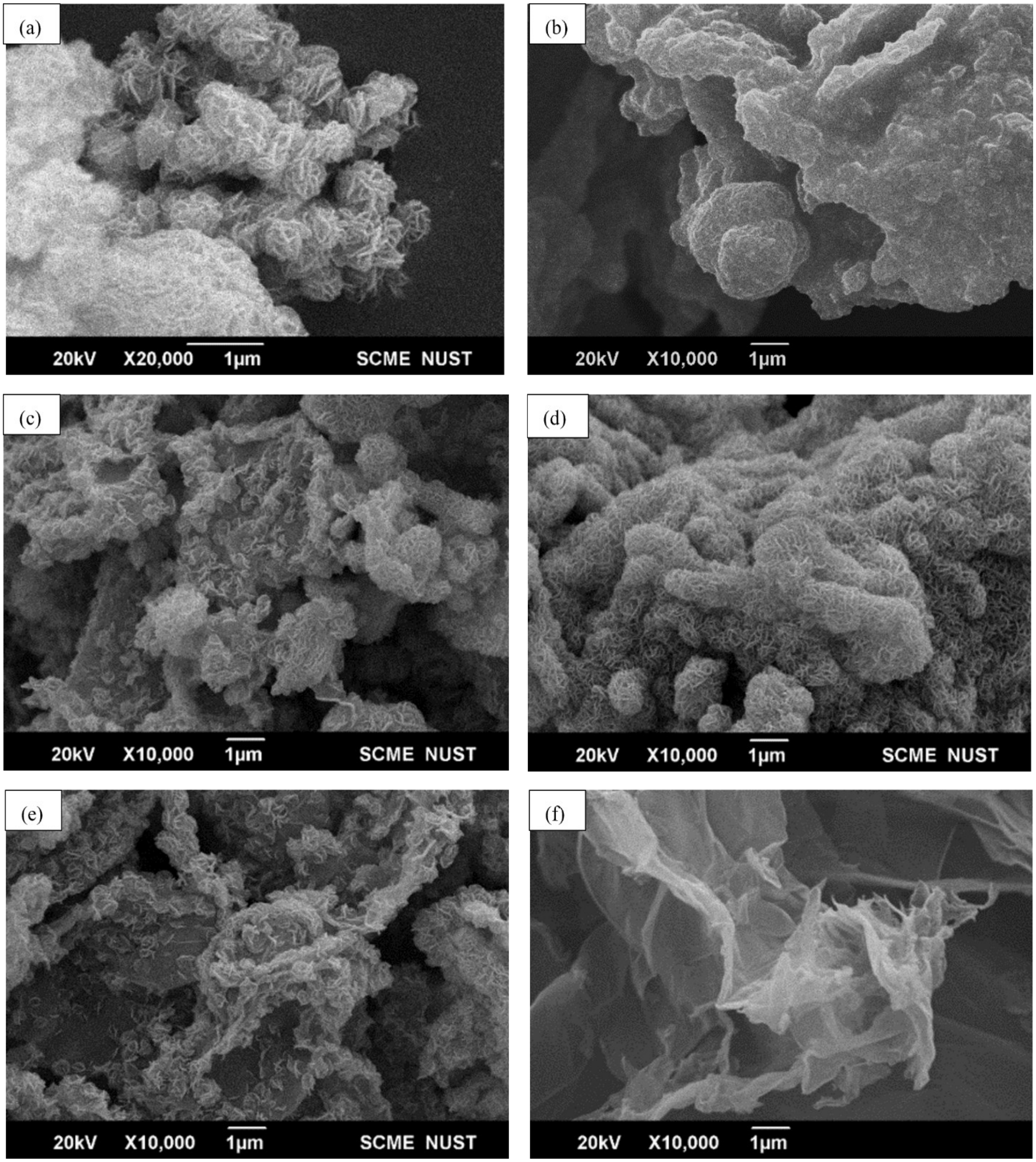

Figure S1: SEM images of (a) Pure MoS_2 (b) 1 wt % MoS_2/rGO (c) 3 wt % MoS_2/rGO (d) 5 wt % MoS_2/rGO (e) 8 wt % MoS_2/rGO (f) rGO sheets

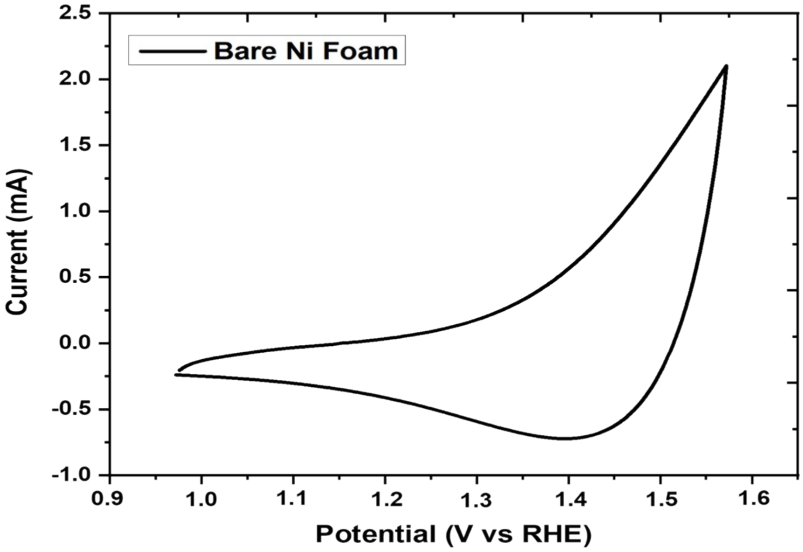

Figure S2. : Cyclic voltammetry of bare nickel foam in 1 M KOH

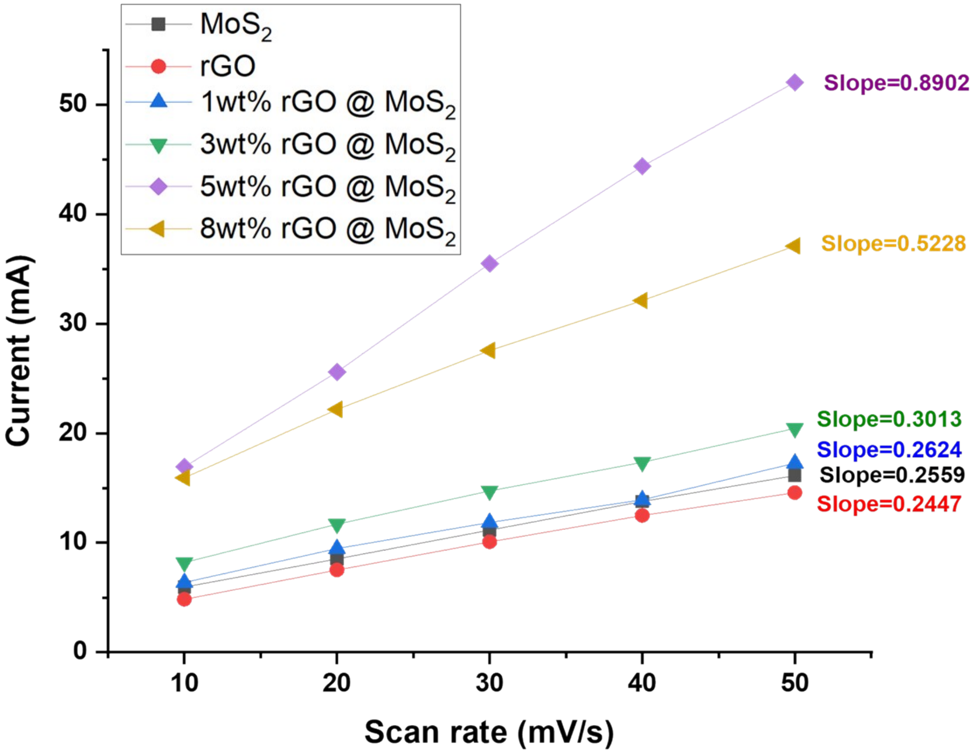

Figure S3: Electrochemical surface comparison for pure MoS_2, rGO, 1, 3, 5 and 8 wt % hybrids

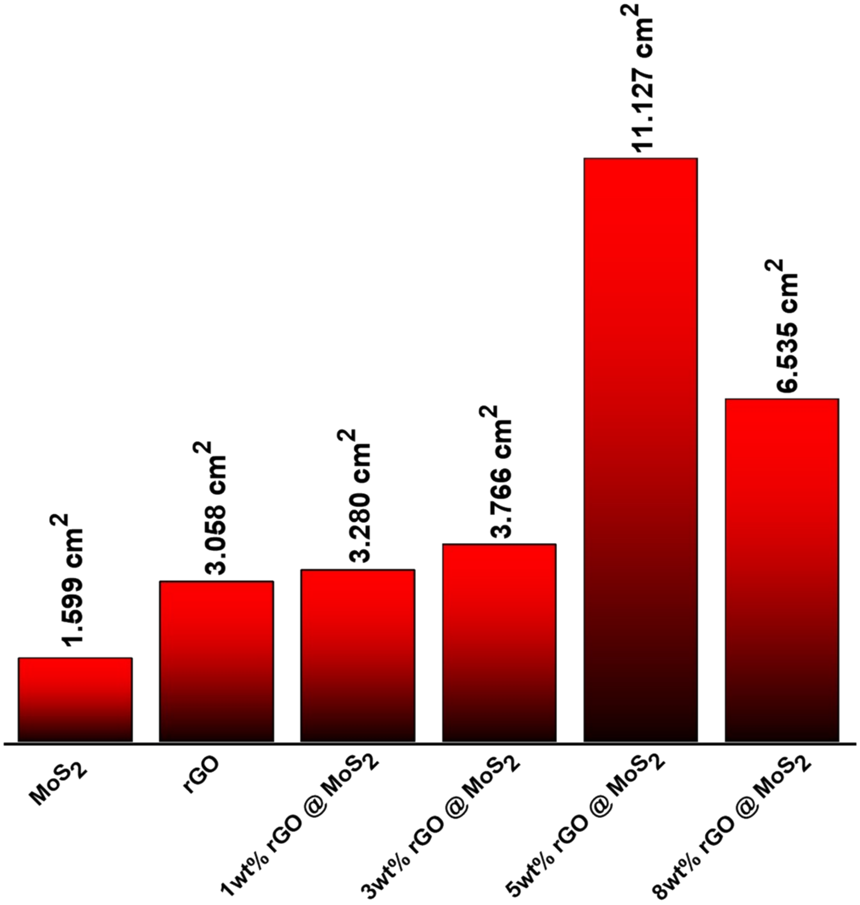

Figure S4: Electrochemical surface Area comparison for pure MoS_2, rGO, 1, 3, 5 and 8 wt % hybrids
